# Perforation and abscess formation after radiological placement of a retrievable plastic biliary stent

**DOI:** 10.1186/1752-1947-5-103

**Published:** 2011-03-14

**Authors:** Ioanna Papadopoulou, Nicos I Fotiadis, Irfan Ahmed, Peter Thurley, Robert R Hutchins, Tim Fotheringham

**Affiliations:** 1 Department of Hepatopancreatobiliary Surgery, Barts and The London NHS Trust, The Royal London Hospital, London, E1 1BB, UK; 2 Department of Diagnostic Imaging, Barts and The London NHS Trust, The Royal London Hospital, London, E1 1BB, UK

## Abstract

**Introduction:**

Retrievable plastic biliary stents are usually inserted endoscopically. When endoscopic placement fails, radiological percutaneous transhepatic placement is indicated. We report the occurrence of a case of delayed duodenal perforation with abscess formation after radiological placement of a plastic stent. To the best of our knowledge, this is the first report of this complication after radiological stenting.

**Case presentation:**

A 58-year-old Caucasian man had a mass 30 mm in size in the head of the pancreas and obstructive jaundice. He was referred for radiological insertion of plastic biliary stents after a failed endoscopic attempt. The procedure was uneventful, and the patient was discharged. Two weeks after the procedure, the patient presented with an acute abdomen and signs of sepsis. Computed tomography revealed erosion of the posterior duodenal wall from the plastic stent, and a large retroperitoneal abscess. The abscess was drained under computed tomography guidance, and the migrated stent was removed percutaneously with a snare under fluoroscopic guidance. Our patient had an uneventful recovery and was discharged after a week.

**Conclusion:**

Late retroperitoneal duodenal perforation is a very rare but severe complication of biliary stenting with plastic stents. Gastroenterologists, surgeons and radiologists should all be aware of its existence, clinical presentation and management.

## Introduction

Complications after endoscopic insertion of retrievable plastic biliary stents are well recognised, including perforation of the duodenum, small bowel and large bowel after migration of the stent [1-4]. Retrievable plastic stents are usually inserted endoscopically, but in our institution are also inserted radiologically when endoscopic placement has failed, and the patient has benign disease or is likely to undergo curative surgery. We report a case of delayed perforation and abscess formation after radiological insertion of a plastic stent. To the best of our knowledge, this is the first report of this complication after radiological stenting. The diagnosis and treatment options are discussed.

## Case presentation

**Figure 1:**
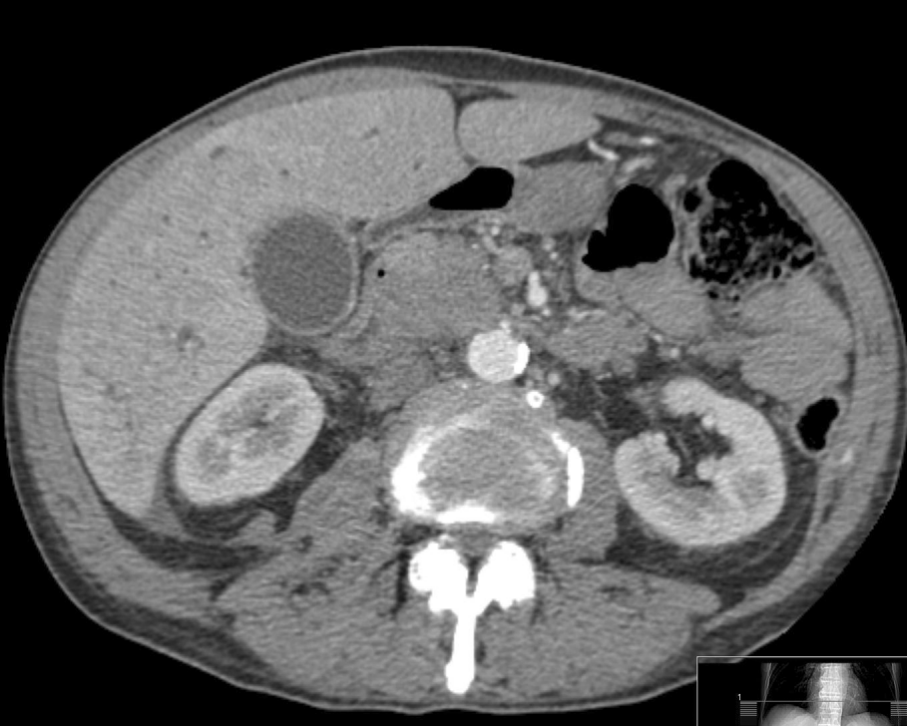
**Axial computed tomography image showing a low-density soft-tissue mass in the head of the pancreas, with a clear fat plane between the mass and the superior mesenteric vein**.

**Figure 2:**
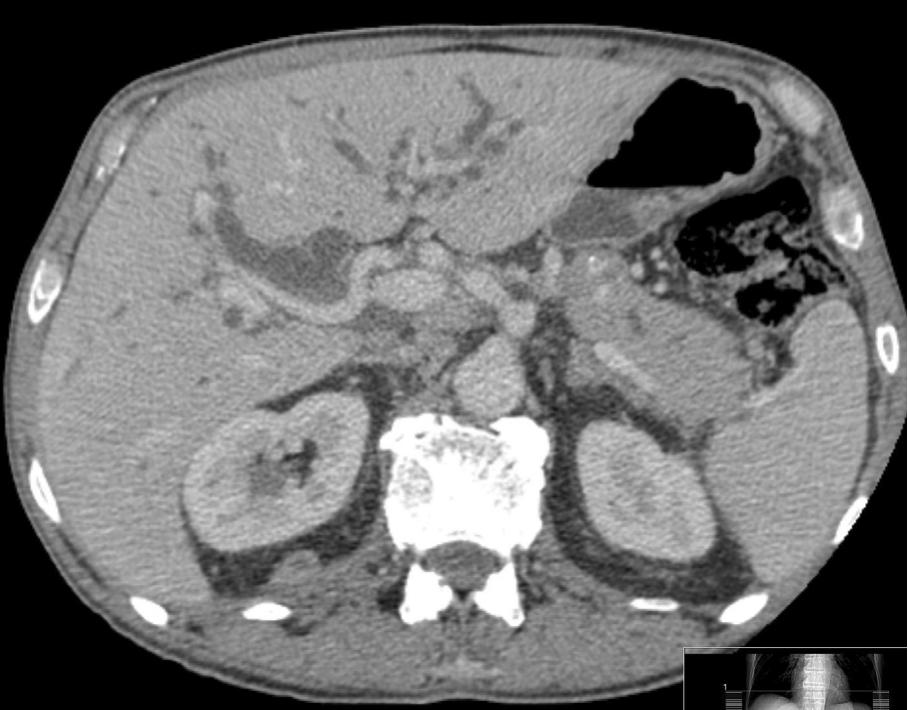
**Axial computed tomography image showing intrahepatic duct dilatation**.

**Figure 3:**
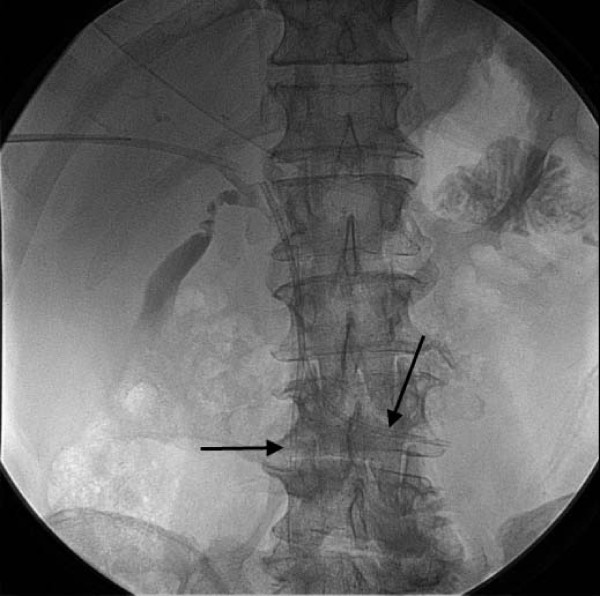
**Single view at the end of percutaneous bilary stenting**. The two plastic biliary stents are seen in satisfactory position at the common bile duct, with the distal tip in the duodenum (black arrows).

A previously well 58-year-old British Caucasian man presented with a three-week history of right upper quadrant pain, vomiting and jaundice. Using computed tomography (CT), a mass 30 mm in size in the head of pancreas was identified, with associated biliary dilatation (Figures 1 and 2). The mass was potentially resectable. It was not possible to cross the biliary obstruction at endoscopic retrograde cholangiopancreatography. As a result, the patient was referred for radiological percutaneous drainage. Under ultrasonography guidance, a right lobe duct was punctured and a catheter was manipulated through the obstruction of the distal common bile duct in the duodenum. A biliary brush cytology sample was obtained. Through a 9Fr peel-away sheath, two 8.5 Fr × 10 mm plastic stents (Cotton-Leung; Cook Medical, Bloomington, IN, USA) were inserted across the obstruction using fluoroscopic guidance. The position of the stents appeared satisfactory (Figure 3), and cholangiography confirmed good drainage. The total bilirubin dropped from 80 μmol/l to 15 μmol/l five days after the procedure and the patient was discharged. The cytology sample confirmed adenocarcinoma.

**Figure 4:**
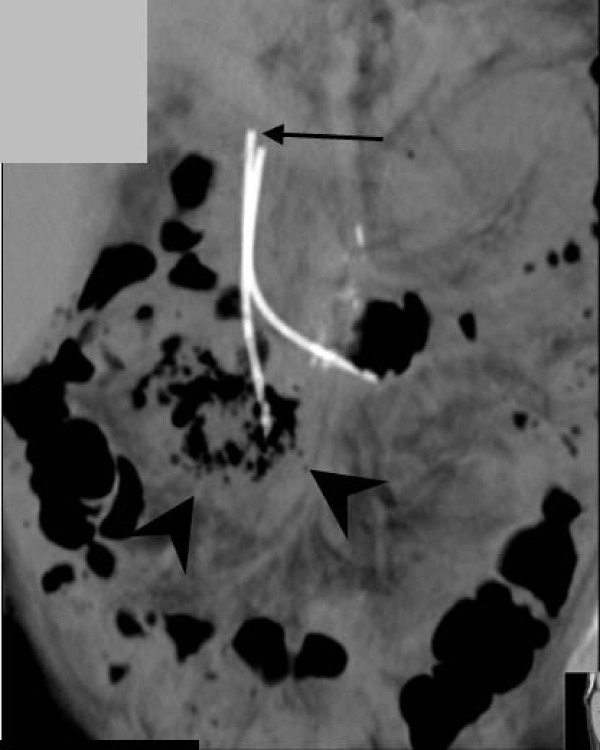
**Coronal reformat computed tomography image**. One of the stents has migrated (white arrow) and its tip is lying in a large, gas-filled retroperitoneal abscess (black arrowheads).

**Figure 5:**
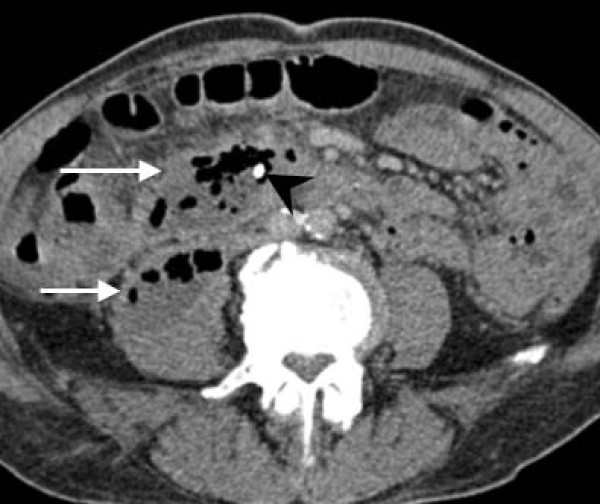
**Axial computed tomography image**. Large, gas-filled retroperitoneal abscess (white arrows) with the tip of the stent inside the collection (arrowhead).

Two weeks after insertion of the stents, the patient developed upper abdominal discomfort and pyrexia, and was admitted for further investigation. On physical examination, right upper-quadrant tenderness with guarding was found. Initial laboratory tests revealed mildly deranged total bilirubin of 20 μmol/l, with slight increase in inflammatory markers and white blood cell count. Abdominal CT was performed the next day, which revealed a large gas-containing collection in the retroperitoneum, with evidence of migration of one of the plastic stents, which appeared to have eroded through the posterior wall of the duodenum (Figures 4, 5). The second stent was in a satisfactory position, allowing biliary drainage. Our patient was started on intravenous piperacillin and tazobactam and amoxicillin and clavulanic acid, with parenteral nutrition. In view of the CT findings, the patient was referred for CT-guided drainage of the collection. The migrated stent was snared under fluoroscopic guidance, and a 12F pigtail drain was inserted into the collection. Frank pus was drained.

Our patient's symptoms resolved and he remained afebrile. Six days after the drainage procedure he reported pain around the site of the drain. On physical examination, pus was found to be leaking from the drain site, and it was decided that the drain should be manipulated under CT guidance. During the procedure, despite the 12F drain being correctly placed, no significant decrease in the size of the localized fluid collection was noted, so two more 8Fr drains were inserted. After one week, there was minimal drainage, so the drains were removed, and our patient discharged. CT performed a month later showed that the remaining stent was in a satisfactory position. The collections identified previously below the level of the duodenum and in the right retroperitoneal region had almost completely resolved.

## Discussion

Endoscopically inserted plastic stents may be used for treatment of both benign biliary obstruction, such as choledocholithiasis, and malignant pathology such as ampullary carcinoma. Occasionally the endoscopist is unable to cross the obstruction, and radiological placement has been described as an alternative [5]. In this case, the endoscopists tried unsuccessfully to cross the obstruction caused by the malignancy before referring the patient for percutaneous radiological plastic stent insertion.

The most common complication associated with insertion of plastic stents in the bile duct is occlusion caused by debris or tumour overgrowth. Other common complications described include cholecystitis, cholangitis, cholestasis, bile-duct erosion and pancreatitis [6]. However, proximal or distal migration of the plastic stent is less common (overall incidence <6%) and is generally not a life-threatening complication unless it causes bowel perforation or obstruction [6]. The risk of stent migration is higher in treatment of benign than malignant diseases, and it has been shown that cases with multiple stents have significantly lower rates of stent migration [7]. Our patient was diagnosed with carcinoma in the head of pancreas. We decided, therefore, to insert two plastic stents to ensure adequate drainage of his biliary tree.

The duodenum is the most common site of perforation of a migrated stent [3,4,6]. Duodenal perforation is most likely to be caused by necrosis of the wall of the duodenum due to the mechanical force exerted by the tip of the plastic stent [4], and can be either intraperitoneal or retroperitoneal. Because of the generalized biliary peritonitis, intraperitoneal duodenal perforation presents earlier and with severe symptoms, whereas retroperitoneal duodenal perforation often presents with mild abdominal discomfort, flank pain, fever, nausea or vomiting, or may even cause no symptoms [6]. Retroperitoneal perforation, therefore, requires an extremely high index of suspicion. Both conditions require immediate management because if not treated, they can very quickly progress to overt sepsis [3,4].

In our case, the stent was noted to have migrated distally, causing a duodenal perforation. Distal bowel perforation due to migration of the stent is an exceedingly rare complication [2]. Ingested foreign bodies generally pass through the lumen of the bowel without causing injury. Patients with coexisting abdominal diseases such as hernias and diverticulae are at higher risk of distal intestinal perforation due to migrated plastic stent [8]. Fistulisation (interenteric or biliocolic, colovaginal or colovesicular) has also been reported [6].

CT is thought to be the most accurate imaging technique for diagnosis of stent problems as it can identify intraperitoneal and retroperitoneal fluid and gas. Treatment options vary, depending on factors such as the patient's stability and age, location of the stent, and presence of coexisting diseases. When the patient is stable, non-operative management with IV antibiotics and parenteral alimentation may be successful. The stent can be retrieved either under fluoroscopic endoscopic guidance or, depending on its location. Stents not accessible to endoscopic- or fluororoscopic-guided retrieval require early operative removal to prevent further complications [9]. Patients with severe clinical symptoms or radiological evidence suggesting extensive contamination will probably require laparotomy [10].

## Conclusion

Duodenal perforation after endoscopic insertion of plastic stents is an infrequent complication that has been well described. We report the first case of duodenal perforation after migration of a plastic stent inserted under radiological guidance. Radiological insertion of plastic stents under fluoroscopic guidance is a useful technique, especially when endoscopic attempts have failed; however, radiologists undertaking this procedure should be aware of this uncommon complication.

## Consent

Written informed consent was obtained from the patient for publication of this case report and accompanying images. A copy of the written consent is available for review by the Editor-in-Chief of this journal.

## Competing interests

The authors declare that they have no competing interests.

## Authors' contributions

IP has a major contribution in writing the manuscript. NF drafted and edited the manuscript. IA performed the percutaneous biliary drainage. PT performed a pecrcutaneous CT guided drainage and assisted with drafting the manuscript. RRH looked after the patient clinically and TF performed the percutaneous retrieval of the migrated stent. All authors read and approved the final manuscript.
